# Supplementary material for: Skin Anti-Inflammatory Potential with Reduced Side Effects of Novel Glucocorticoid Receptor Agonists
Source: Int J Mol Sci. 2023 Dec 23;25(1):267. doi: 10.3390/ijms25010267 (PMC10778823; doi:10.3390/ijms25010267)
Supplement: Supplementary file 1 [file ijms-25-00267-s001.zip › ijms-2758150-supplementary.pdf]

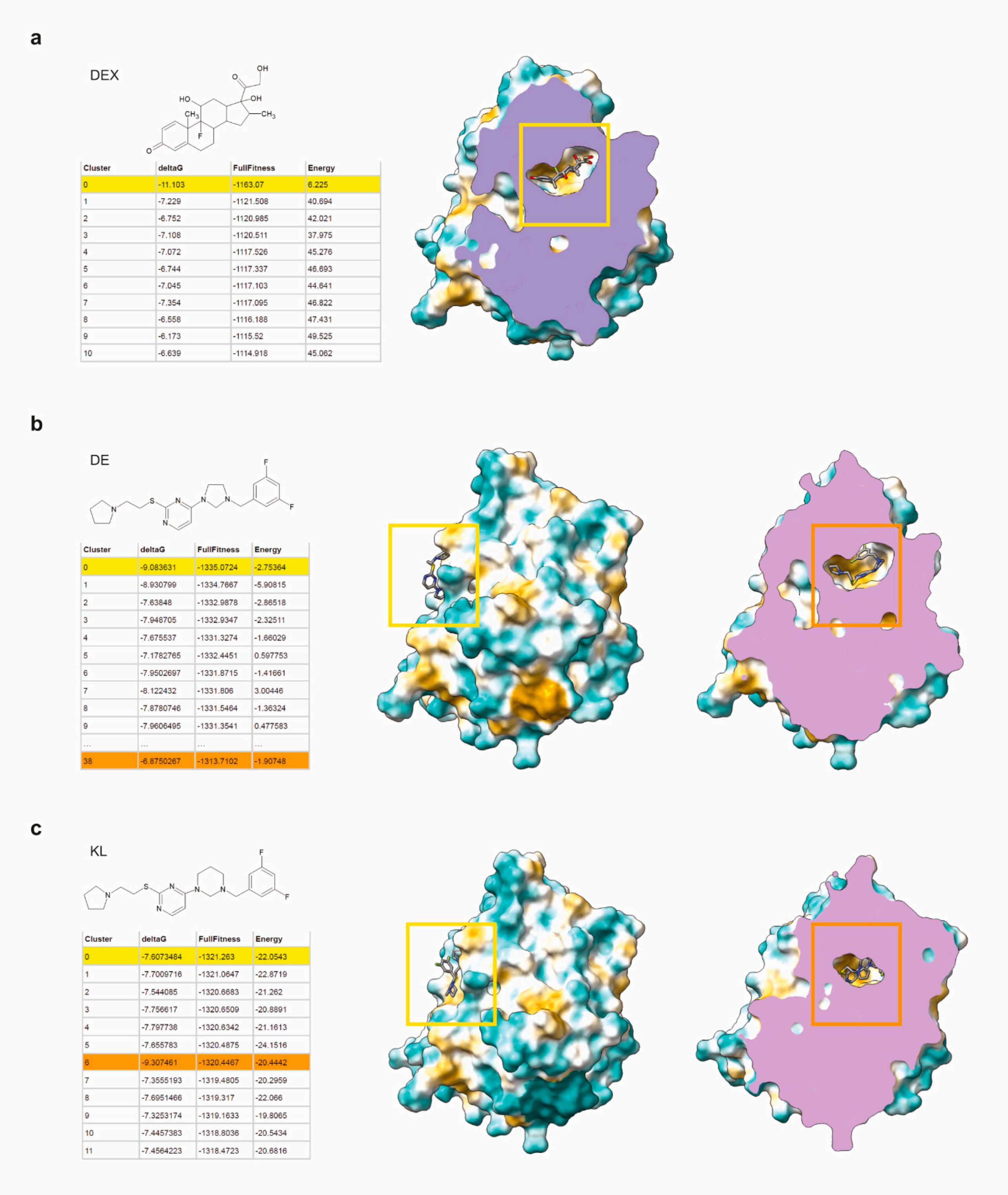

Cluster	deltaG	FullFitness	Energy
0	-11.103	-1163.07	6.225
1	-7.229	-1121.508	40.694
2	-6.752	-1120.985	42.021
3	-7.108	-1120.511	37.975
4	-7.072	-1117.526	45.276
5	-6.744	-1117.337	46.693
6	-7.045	-1117.103	44.641
7	-7.354	-1117.095	46.822
8	-6.558	-1116.188	47.431
9	-6.173	-1115.52	49.525
10	-6.639	-1114.918	45.062

Cluster	deltaG	FullFitness	Energy
0	-9.083631	-1335.0724	-2.75364
1	-8.930799	-1334.7667	-5.90815
2	-7.63848	-1332.9878	-2.86518
3	-7.948705	-1332.9347	-2.32511
4	-7.675537	-1331.3274	-1.66029
5	-7.1782765	-1332.4451	0.597753
6	-7.9502697	-1331.8715	-1.41661
7	-8.122432	-1331.806	3.00446
8	-7.8780746	-1331.5464	-1.36324
9	-7.9606495	-1331.3541	0.477583
...	...	...	...
38	-6.8750267	-1313.7102	-1.90748

Cluster	deltaG	FullFitness	Energy
0	-7.6073484	-1321.263	-22.0543
1	-7.7009716	-1321.0647	-22.8719
2	-7.544085	-1320.6683	-21.262
3	-7.756617	-1320.6509	-20.8891
4	-7.797738	-1320.6342	-21.1613
5	-7.655783	-1320.4875	-24.1516
6	-9.307461	-1320.4467	-20.4442
7	-7.3555193	-1319.4805	-20.2959
8	-7.6951466	-1319.317	-22.066
9	-7.3253174	-1319.1633	-19.8065
10	-7.4457383	-1318.8036	-20.5434
11	-7.4564223	-1318.4723	-20.6816

Figure S1. Chemical structure and molecular docking of DE and KL. Schematic representation of the chemical structure of (**a**) Dexamethasone, (**b**) DE, and (**c**) KL, and their 3D molecular model of docking to GC receptor.

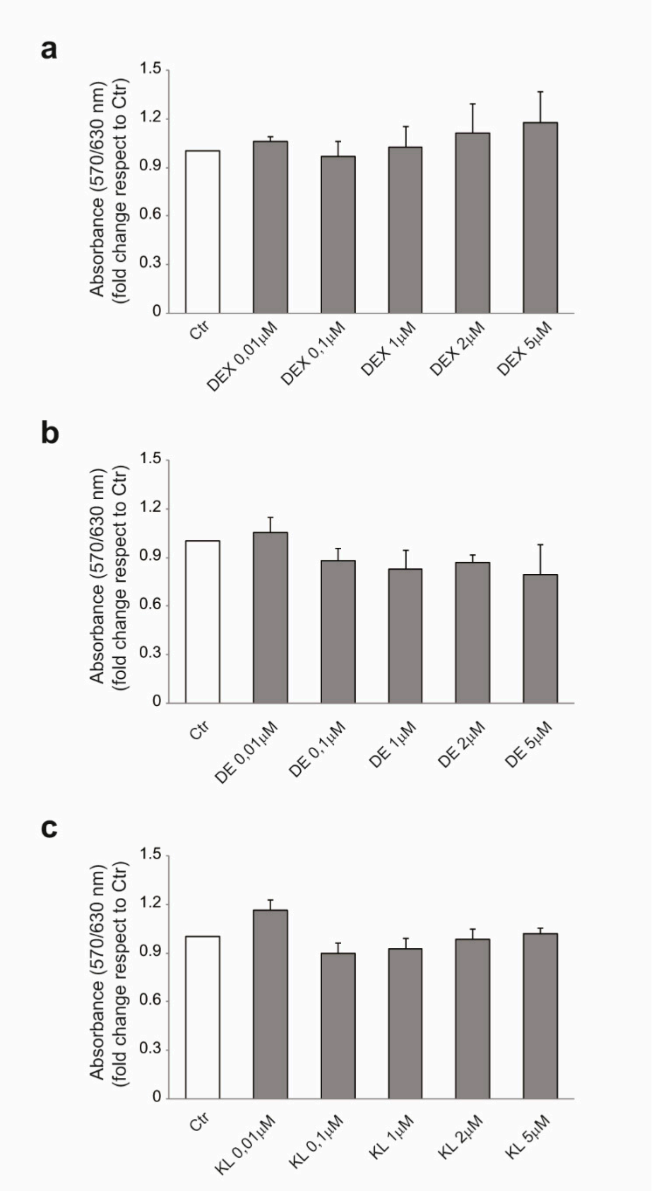

Figure S2. Effect of DE and KL on NHK viability. MTT assay in NHKs treated with (**a**) DEX, (**b**) DE, and (**c**) KL at the dose range of 0.01-5 μM for 72 hours.

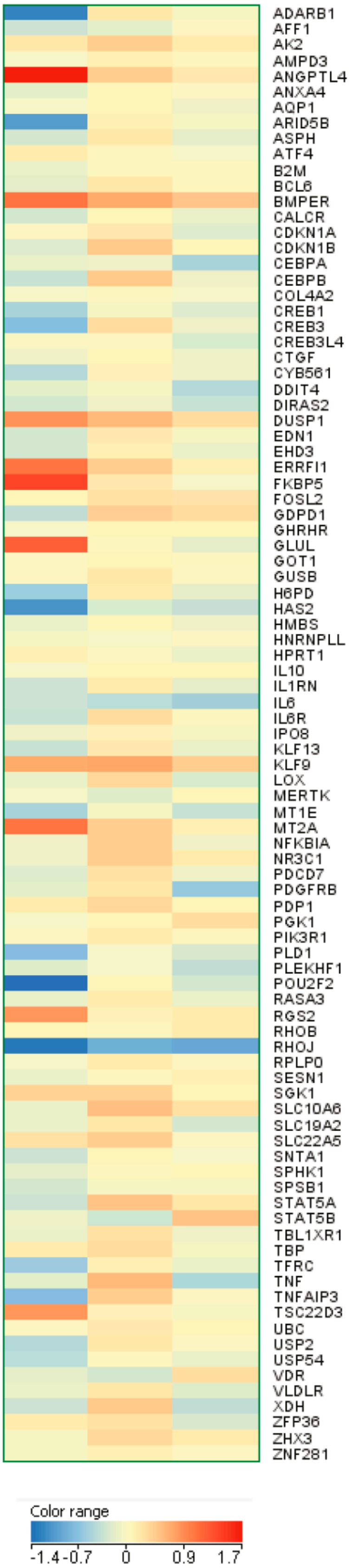

Figure S3. High magnification of array card heatmap. Array card heatmap illustrating gene expression analysis in NHKs treated with DEX, DE, and KL (1μM) for 24 hours. Red and blue shadings represent higher and lower relative log2 fold change expression levels, respectively.

Table S1. Forward (F) and reverse (R) primers used for the Real time RT-PCR analysis.

Gene	Oligonucleotide Sequences (5′-3′)	Amplicon Size	Accession Number
ANGPTL4	F: CGACAACAAGTACAAGAT<br>R: CTCGTTAGATGTCCAAAG	107 bp	NM_001322809.1
COL1A1	F: CAGCCGCTTCACCTACAGC<br>R: AATCACTGTCTTGCCCCAGG	73 bp	NM_000088.4
COL3A1	F: TCCAACTGCTCCTACTCGCC<br>R: GAGGGCCTGGATCTCCCTT	68 bp	NM_000090.4
DUSP1	F: GATCAACGTCTCAGCCAATT<br>R: CTATGAAGTCAATGGCCTCG	125 bp	NM_004417.3
ERRFI1	F: GTTCCCATACCTCCTAGACC<br>R: TACTCGGTGACAAAGGTTCT	127 bp	NM_018948.3
FKBP5	F: TGTCCTGGTTAGAGATGGAA<br>R: CTTGTCACAGCATTCAACAG	143 bp	NM_005953.4
GLUL	F: TGAGAAACTAAGCAAGCGG<br>R: TACACCAGCAGAAAAGTCG	129 bp	NM_002065.6
HAS2	F: CCTCATCATCCAAAGCCTGT<br>R: GATGCAAAGGGCAACTGTTT	100 bp	NM_005328.3
HAS3	F: GTCAGTGGTCACGGGTTTCT<br>R: GACACAGGAATGAGGCCAAT	309 bp	NM_005329.3
MMP1	F: GGGAGATCATCGGGACAACTC<br>R: CCAATACCTGGGCCTGGTTGA	81 bp	NM_002421.4
MMP2	F: AGAAGGCTGTGTTCTTTGCAG<br>R: AGGCTGGTCAGTGGCTTG	88 bp	NM_004530.6
MMP3	F: GCTGCAAGGGGTGAGGACAC<br>R: GATGCCAGGAAAGGTTCTGAAGTG	252 bp	NM_002422.5
MT2A	F: GTACAACCCTGACCGTGACC<br>R: TCACATTATTTCATAGAAAAAGGAATATAGC	56 bp	NM_001145775.2
RGS2	F: CCTCAAAAGCAAGGAAAATATATACTGA<br>R: AGTTGTAAAGCAGCCACTTGTAGCT	131 bp	NM_002923.3
TSC22D3	F: GATGTGGTTTCCGTTAAGCT<br>R: CTCCTCTCTCACAGCATACA	123 bp	NM_004089.3

Table S2. MRM transitions for the analysis of ceramide species by ESI-MS/MS.

Species	Precursor Ion	Product Ion	Fragmentor (V)	Collision Energy (eV)	Polarity
ADS	**$[M+H-H2O]^+$**	**$[M+H-FA-2H_2O]^+$**			
**A(14)DS(24)**	594.6	350	135	30	positive
**A(15)DS(16)**	496.5	238	135	30	positive
**A(15)DS(18)**	524.5	266	135	30	positive
**A(15)DS(19)**	538.5	280	135	30	positive
**A(16)DS(16)**	510.5	238	135	30	positive
**A(16)DS(18)**	538.5	266	135	30	positive
**A(16)DS(19)**	552.5	280	135	30	positive
**A(16)DS(20)**	566.6	294	135	30	positive
**A(16)DS(21)**	580.6	308	135	30	positive
**A(17)DS(18)**	552.5	266	135	30	positive
**A(18)DS(16)**	538.5	238	135	30	positive
**A(18)DS(18)**	566.6	266	135	30	positive
**A(18)DS(20)**	594.6	294	135	30	positive
**A(18)DS(23)**	636.6	336	135	30	positive
**A(18)DS(24)**	650.6	350	135	30	positive
**A(19)DS(16)**	552.5	238	135	30	positive
**A(19)DS(18)**	580.6	266	135	30	positive
**A(19)DS(22)**	636.6	322	135	30	positive
**A(20)DS(16)**	566.6	238	135	30	positive
**A(20)DS(17)**	580.6	252	135	30	positive
**A(20)DS(18)**	594.6	266	135	30	positive
**A(21)DS(18)**	608.6	266	135	30	positive
**A(22)DS(17)**	608.6	252	135	30	positive
**A(22)DS(18)**	622.6	266	135	30	positive
**A(22)DS(20)**	650.6	294	135	30	positive
**A(22)DS(21)**	664.7	308	135	30	positive
**A(22)DS(24)**	706.7	350	135	30	positive
**A(23)DS(18)**	636.6	266	135	30	positive
**A(23)DS(19)**	650.6	280	135	30	positive
**A(23)DS(22)**	692.7	322	135	30	positive
**A(24)DS(18)**	650.6	266	135	30	positive
**A(24)DS(19)**	664.7	280	135	30	positive
**A(24)DS(20)**	678.6	294	135	30	positive
**A(24)DS(21)**	682.7	308	135	30	positive
**A(25)DS(17)**	650.6	252	135	30	positive
**A(25)DS(18)**	664.7	266	135	30	positive
**A(26)DS(16)**	650.6	238	135	30	positive
**A(26)DS(17)**	664.7	252	135	30	positive
**A(26)DS(18)**	678.7	266	135	30	positive

**A(27)DS(18)**	692.7	266	135	30	positive
**A(28)DS(18)**	706.7	266	135	30	positive
AP	**[M+H-H2O]+**	**[M+H-FA-2H2O]+**			
**A(14)P(21)**	568.5	324	135	30	positive
**A(14)P(22)**	582.5	338	135	30	positive
**A(14)P(24)**	610.6	366	135	30	positive
**A(15)P(20)**	568.5	310	135	30	positive
**A(15)P(21)**	582.6	324	135	30	positive
**A(15)P(22)**	596.6	338	135	30	positive
**A(16)P(18)**	554.5	282	135	30	positive
**A(17)P(17)**	554.5	268	135	30	positive
**A(17)P(18)**	568.5	282	135	30	positive
**A(17)P(21)**	610.6	324	135	30	positive
**A(17)P(23)**	638.6	352	135	30	positive
**A(18)P(16)**	554.5	254	135	30	positive
**A(18)P(18)**	582.6	282	135	30	positive
**A(18)P(20)**	610.6	310	135	30	positive
**A(18)P(21)**	624.6	324	135	30	positive
**A(18)P(22)**	638.6	338	135	30	positive
**A(18)P(23)**	652.6	352	135	30	positive
**A(18)P(24)**	666.7	366	135	30	positive
**A(20)P(18)**	610.6	282	135	30	positive
**A(21)P(18)**	624.6	282	135	30	positive
**A(21)P(21)**	666.7	324	135	30	positive
**A(21)P(24)**	710.7	366	135	30	positive
**A(22)P(18)**	638.6	282	135	30	positive
**A(22)P(20)**	666.7	310	135	30	positive
**A(23)P(16)**	624.6	254	135	30	positive
**A(23)P(18)**	652.6	282	135	30	positive
**A(24)P(18)**	666.7	282	135	30	positive
**A(24)P(21)**	708.7	324	135	30	positive
**A(25)P(18)**	680.7	282	135	30	positive
**A(25)P(20)**	708.7	310	135	30	positive
**A(26)P(18)**	694.7	282	135	30	positive
**A(26)P(19)**	708.7	296	135	30	positive
**A(27)P(17)**	694.7	268	135	30	positive
**A(27)P(18)**	708.7	282	135	30	positive
AS	**[M+H-H2O]+**	**[M+H-FA-2H2O]+**			
**A(14)S(16)**	480.5	236	135	30	positive
**A(14)S(18)**	508.5	264	135	30	positive
**A(14)S(20)**	536.5	292	135	30	positive
**A(14)S(21)**	550.5	306	135	30	positive
**A(14)S(22)**	564.6	320	135	30	positive

**A(14)S(23)**	578.6	334	135	30	positive
**A(14)S(24)**	592.6	348	135	30	positive
**A(15)S(23)**	592.6	334	135	30	positive
**A(16)S(18)**	536.5	264	135	30	positive
**A(17)S(16)**	522.5	236	135	30	positive
**A(17)S(18)**	550.5	264	135	30	positive
**A(17)S(21)**	592.6	306	135	30	positive
**A(17)S(24)**	634.6	348	135	30	positive
**A(18)S(16:2)**	534.5	234	135	30	positive
**A(18)S(18)**	564.6	264	135	30	positive
**A(18)S(18:2)**	562.6	262	135	30	positive
**A(18)S(19)**	578.6	278	135	30	positive
**A(18)S(23)**	634.6	334	135	30	positive
**A(18)S(24)**	648.6	348	135	30	positive
**A(18:1)S(16)**	534.5	236	135	30	positive
**A(18:1)S(16:2)**	532.5	234	135	30	positive
**A(19)S(18)**	578.6	264	135	30	positive
**A(19)S(23)**	648.6	334	135	30	positive
**A(19)S(24)**	662.7	348	135	30	positive
**A(20)S(16)**	564.6	236	135	30	positive
**A(20)S(16:2)**	562.6	234	135	30	positive
**A(20)S(17)**	578.6	250	135	30	positive
**A(20)S(18)**	592.6	264	135	30	positive
**A(20)S(18:2)**	590.6	262	135	30	positive
**A(20)S(19)**	606.6	278	135	30	positive
**A(20)S(21)**	634.6	306	135	30	positive
**A(20)S(24)**	676.7	348	135	30	positive
**A(20:1)S(16)**	562.5	236	135	30	positive
**A(20:1)S(16:2)**	560.5	234	135	30	positive
**A(21)S(21)**	648.6	306	135	30	positive
**A(21)S(22)**	662.7	320	135	30	positive
**A(21)S(23)**	676.7	334	135	30	positive
**A(22)S(16)**	592.6	236	135	30	positive
**A(22)S(16:2)**	590.6	232	135	30	positive
**A(22)S(20)**	648.6	292	135	30	positive
**A(22)S(21)**	662.7	306	135	30	positive
**A(22:1)S(16:2)**	588.6	234	135	30	positive
**A(23)S(18)**	634.6	264	135	30	positive
**A(24)S(16:2)**	618.6	232	135	30	positive
**A(24)S(18)**	648.6	264	135	30	positive
**A(24)S(20)**	676.7	292	135	30	positive
**A(25)S(16)**	634.6	236	135	30	positive
**A(25)S(17)**	648.6	250	135	30	positive
**A(25)S(18)**	662.7	264	135	30	positive
**A(26)S(16)**	648.6	236	135	30	positive
**A(26)S(17)**	662.7	250	135	30	positive

**A(26)S(18)**	676.7	264	135	30	positive
**A(27)S(16)**	662.7	236	135	30	positive
**A(28)S(16)**	676.7	236	135	30	positive
NDS	**[M+H-H2O]+**	**[M+H-FA-2H2O]+**			
**N(13)DS(18)**	480.5	266	135	30	positive
**N(14)DS(16)**	466.5	238	135	30	positive
**N(14)DS(18)**	494.5	266	135	30	positive
**N(14)DS(24)**	578.6	350	135	30	positive
**N(15)DS(18)**	508.5	266	135	30	positive
**N(16)DS(16)**	494.5	238	135	30	positive
**N(16)DS(17)**	508.5	252	135	30	positive
**N(16)DS(18)**	522.5	266	135	30	positive
**N(16)DS(22)**	578.6	322	135	30	positive
**N(16)DS(24)**	606.6	350	135	30	positive
**N(17)DS(16)**	508.5	238	135	30	positive
**N(17)DS(18)**	536.5	266	135	30	positive
**N(17)DS(24)**	620.6	350	135	30	positive
**N(18)DS(17)**	536.5	252	135	30	positive
**N(18)DS(18)**	550.6	266	135	30	positive
**N(18)DS(19)**	564.6	280	135	30	positive
**N(18)DS(20)**	578.6	294	135	30	positive
**N(18)DS(22)**	606.6	322	135	30	positive
**N(18)DS(24)**	634.6	350	135	30	positive
**N(18:1)DS(18)**	548.6	266	135	30	positive
**N(19)DS(19)**	578.6	280	135	30	positive
**N(19)DS(20)**	592.6	294	135	30	positive
**N(19)DS(23)**	634.6	336	135	30	positive
**N(19)DS(24)**	646.7	350	135	30	positive
**N(20)DS(16)**	550.6	238	135	30	positive
**N(20)DS(18)**	578.6	266	135	30	positive
**N(20)DS(22)**	632.6	322	135	30	positive
**N(20)DS(23)**	648.7	336	135	30	positive
**N(20)DS(24)**	662.7	350	135	30	positive
**N(21)DS(16)**	564.6	238	135	30	positive
**N(21)DS(17)**	578.6	252	135	30	positive
**N(21)DS(18)**	592.6	266	135	30	positive
**N(21)DS(19)**	606.6	280	135	30	positive
**N(21)DS(20)**	620.6	294	135	30	positive
**N(21)DS(22)**	648.7	322	135	30	positive
**N(21)DS(24)**	676.7	350	135	30	positive
**N(22)DS(16)**	578.6	238	135	30	positive
**N(22)DS(17)**	592.6	252	135	30	positive
**N(22)DS(18)**	606.6	266	135	30	positive
**N(22)DS(20)**	634.6	294	135	30	positive

**N(22)DS(24)**	690.7	350	135	30	positive
**N(23)DS(16)**	592.6	238	135	30	positive
**N(23)DS(18)**	620.6	266	135	30	positive
**N(24)DS(17)**	620.6	252	135	30	positive
**N(24)DS(20)**	662.7	294	135	30	positive
**N(24)DS(22)**	690.7	322	135	30	positive
**N(25)DS(21)**	690.7	308	135	30	positive
**N(26)DS(16)**	634.6	238	135	30	positive
**N(26)DS(17)**	648.7	252	135	30	positive
**N(26)DS(18)**	676.7	280	135	30	positive
**N(26)DS(19)**	662.7	266	135	30	positive
**N(27)DS(16)**	648.7	238	135	30	positive
**N(27)DS(17)**	662.7	252	135	30	positive
**N(27)DS(18)**	676.7	266	135	30	positive
**N(28)DS(16)**	662.7	238	135	30	positive
**N(28)DS(17)**	676.7	252	135	30	positive
**N(28)DS(18)**	690.7	266	135	30	positive
**N(29)DS(16)**	676.7	238	135	30	positive
**N(29)DS(17)**	690.7	252	135	30	positive
**N(30)DS(16)**	690.7	238	135	30	positive
NP	**$[M+H-H2O]^+$**	**$[M+H-FA-2H_2O]^+$**			
**N(14)P(17)**	496.5	268	135	30	positive
**N(14)P(18)**	510.5	282	135	30	positive
**N(14)P(20)**	538.5	310	135	30	positive
**N(14)P(21)**	552.5	324	135	30	positive
**N(15)P(16)**	496.5	254	135	30	positive
**N(15)P(17)**	510.5	268	135	30	positive
**N(15)P(18)**	524.5	282	135	30	positive
**N(16)P(16)**	510.5	254	135	30	positive
**N(16)P(17)**	524.5	268	135	30	positive
**N(16)P(18)**	538.5	282	135	30	positive
**N(16)P(20)**	566.6	310	135	30	positive
**N(16)P(22)**	594.6	338	135	30	positive
**N(16)P(24)**	622.6	366	135	30	positive
**N(17)P(18)**	552.5	282	135	30	positive
**N(17)P(24)**	636.6	366	135	30	positive
**N(18)P(16)**	538.5	254	135	30	positive
**N(18)P(18)**	566.6	282	135	30	positive
**N(18)P(20)**	594.6	310	135	30	positive
**N(18)P(21)**	608.6	324	135	30	positive
**N(19)P(18)**	580.6	282	135	30	positive
**N(20)P(16)**	566.6	254	135	30	positive
**N(20)P(18)**	594.6	282	135	30	positive
**N(20)P(19)**	608.6	296	135	30	positive

**N(20)P(22)**	650.7	338	135	30	positive
**N(20)P(24)**	678.7	366	135	30	positive
**N(21)P(17)**	594.6	268	135	30	positive
**N(21)P(18)**	608.6	282	135	30	positive
**N(21)P(19)**	622.6	296	135	30	positive
**N(21)P(20)**	636.6	310	135	30	positive
**N(21)P(21)**	650.7	324	135	30	positive
**N(21)P(23)**	678.7	352	135	30	positive
**N(21)P(24)**	692.7	366	135	30	positive
**N(22)P(17)**	608.6	268	135	30	positive
**N(22)P(18)**	622.6	282	135	30	positive
**N(22)P(21)**	664.7	324	135	30	positive
**N(22)P(22)**	678.7	338	135	30	positive
**N(22)P(24)**	706.7	366	135	30	positive
**N(23)P(18)**	636.6	282	135	30	positive
**N(23)P(21)**	678.7	324	135	30	positive
**N(24)P(18)**	650.7	282	135	30	positive
**N(24)P(20)**	678.7	310	135	30	positive
**N(24:1)P(18)**	648.7	282	135	30	positive
**N(25)P(20)**	692.7	310	135	30	positive
**N(25)P(21)**	706.7	324	135	30	positive
**N(26)P(16)**	650.7	254	135	30	positive
**N(26)P(18)**	678.7	282	135	30	positive
**N(27)P(16)**	664.7	254	135	30	positive
**N(27)P(18)**	692.7	282	135	30	positive
**N(28)P(16)**	678.7	254	135	30	positive
**N(28)P(17)**	692.7	268	135	30	positive
**N(28)P(18)**	706.7	282	135	30	positive
NS	**[M+H-H2O]$^+$**	**[M+H-FA-2H_2O]$^+$**			
**N(14)S(16)**	464.5	236	135	30	positive
**N(14)S(18)**	492.5	264	135	30	positive
**N(15)S(18)**	506.5	264	135	30	positive
**N(16)S(17)**	506.5	250	135	30	positive
**N(16)S(18)**	520.5	264	135	30	positive
**N(16)S(21)**	562.6	306	135	30	positive
**N(17)S(16)**	506.5	236	135	30	positive
**N(17)S(18)**	534.5	264	135	30	positive
**N(18)S(16)**	520.5	236	135	30	positive
**N(18)S(18)**	548.5	264	135	30	positive
**N(18)S(19)**	562.6	278	135	30	positive
**N(18)S(20)**	576.6	292	135	30	positive
**N(18)S(23)**	618.6	334	135	30	positive
**N(18:1)S(18)**	546.5	264	135	30	positive
**N(18:2)S(18)**	544.5	264	135	30	positive

**N(19)S(18)**	562.6	264	135	30	positive
**N(20)S(16)**	548.5	236	135	30	positive
**N(20)S(18)**	576.6	264	135	30	positive
**N(20)S(18:2)**	574.6	262	135	30	positive
**N(20)S(23)**	646.7	334	135	30	positive
**N(20:1)S(16)**	546.5	236	135	30	positive
**N(20:1)S(16:2)**	544.5	234	135	30	positive
**N(20:1)S(18:2)**	572.5	262	135	30	positive
**N(21)S(18:2)**	588.5	262	135	30	positive
**N(22)S(16)**	576.6	236	135	30	positive
**N(22)S(18)**	604.6	264	135	30	positive
**N(22)S(20)**	632.6	292	135	30	positive
**N(22:1)S(16)**	574.6	236	135	30	positive
**N(22)S(18:2)**	602.6	262	135	30	positive
**N(23)S(18)**	618.6	264	135	30	positive
**N(23)S(18:2)**	616.6	262	135	30	positive
**N(23)S(23)**	688.7	334	135	30	positive
**N(24)S(16)**	604.6	236	135	30	positive
**N(24)S(16:2)**	602.6	234	135	30	positive
**N(24)S(17)**	618.6	250	135	30	positive
**N(24)S(18)**	632.6	264	135	30	positive
**N(24:1)S(18)**	630.6	264	135	30	positive
**N(26)S(17)**	646.7	250	135	30	positive
**N(26)S(18)**	660.7	264	135	30	positive
**N(27)S(16)**	646.7	236	135	30	positive
**N(27)S(17)**	660.7	250	135	30	positive
**N(27)S(18)**	674.7	264	135	30	positive
**N(28)S(18)**	688.7	264	135	30	positive
**N(29)S(18)**	702.7	264	135	30	positive
**N(30)S(18)**	716.7	264	135	30	positive
